# Respiratory microbiota transplantation: optimized framework and its impact on metabolic and immune characteristics

**DOI:** 10.1016/j.pccm.2026.05.002

**Published:** 2026-06-08

**Authors:** Shifen Xu, Xing Zhang, Yunfeng Shi, Xiang Tan, Hanqin Cai, Wei Cheng, Lei Yang, Xinzhu Yi, Zhiming Xiang, Chao Cao, Hong Wei, Zhang Wang

**Affiliations:** a Guangdong Engineering Research Center of Precision Detection and Modulation of Human Microbiome; School of Life Sciences, South China Normal University, Guangzhou, Guangdong 510631, China; b Yu-Yue Pathology Scientific Research Center, Jinfeng Laboratory, Chongqing 401329, China; c College of Animal Sciences and Technology, Huazhong Agricultural University, Wuhan, Hubei 430070, China; d Department of Radiology, The Affiliated Panyu Central Hospital of Guangzhou Medical University, Guangzhou, Guangdong 511400, China; e Department of Respiratory and Critical Medicine, Key Laboratory of Respiratory Disease of Ningbo, The First Affiliated Hospital of Ningbo University, Ningbo, Zhejiang 315010, China; f Guangdong Engineering Research Center of Precision Detection and Modulation of Human Microbiome, School of Life Sciences, State Key Laboratory of Respiratory Disease, South China Normal University, Guangzhou, Guangdong 510631, China

**Keywords:** Respiratory microbiome, Microbiota transplantation, Multi-omics, Germ-free mice

## Abstract

**Background:**

The respiratory microbiota is critical to maintaining local immune homeostasis and respiratory health. Microbiota transplantation has proven transformative in gut microbiome studies. However, a standardized approach for the respiratory tract remains lacking, hindered by technical difficulty of establishing a recipient airway niche conducive to stable donor microbial engraftment, and limited systematic evaluation of key parameters that influence transplantation efficacy. This study aims to establish an optimized respiratory microbiota transplantation (RMT) framework and determine whether RMT reshapes metabolic and immunological characteristics.

**Methods:**

We developed and optimized a method for RMT in murine models. Key parameters, including sample storage, delivery route, and treatment regimen, were systematically evaluated for their effects on the microbiome using 16S ribosomal RNA gene sequencing-based profiling. The influence of microbiota transplantation on host metabolism and immunity was also assessed through metabolomic and transcriptomic characterization. Wilcoxon rank-sum test was used to compare Bray-Curtis dissimilarity between groups. Additionally, a one-sample Wilcoxon signed-rank test was used to determine whether the relative abundance changes within each recipient–donor pair significantly deviated from zero. Differential metabolomic and transcriptomic features were identified using trend analysis.

**Results:**

We established mouse-to-mouse RMT by transferring bronchoalveolar lavage fluid (BALF)-derived microbial communities from specific pathogen-free (SPF) donors to germ-free (GF) recipients. To model lung dysbiosis, we induced sepsis via cecal ligation and puncture in SPF mice and transplanted their BALF microbiota into normal SPF recipients. The highest compatibility of donor–recipient microbiota was observed using glycerol-preserved samples, delivered either intratracheally or intranasally every other day, for a duration of 14 days (Wilcoxon rank-sum test, SPF–GF mice: intratracheal delivery 7 days *vs.* 14 days, *W* = 12.0, *P* = 0.057, intranasal delivery 7 days *vs.* 14 days, *W* = 12.0, *P* = 0.057; CLP–SPF mice: intratracheal delivery 7 days *vs.* 14 days, *W* = 35.0, *P* = 0.003, intranasal delivery 7 days *vs.* 14 days: *W* = 36.0, *P* = 0.035). This microbiota transplantation partially shifted the metabolomic and immunological characteristics of GF recipients toward those of SPF donors, reversing 188 metabolites and 2721 host genes that were altered in GF mice compared with SPF mice. We then adapted this protocol for human-to-mouse RMT, transplanting microbiota from human BALF and sputum into SPF mice. Intratracheal and intranasal delivery of human BALF yielded comparable donor–recipient microbiota similarity. However, intratracheal administration significantly increased donor–recipient similarity when sputum-derived microbiota were transplanted (Wilcoxon rank-sum test, *W* = 53.5, *P* = 0.004).

**Conclusion:**

Our study establishes an optimized protocol for RMT using glycerol-preserved samples, delivered either intratracheally or intranasally every other day over 14 days. This approach should empower preclinical investigation of respiratory microbiota and pave the way for clinical translation.

## Introduction

The role of the microbiome in human health and disease has been well established over the past decades. Dysbiosis, disruption of the microbiome, has been implicated in a wide array of metabolic, cardiovascular, respiratory, and neurological disorders, underscoring the need for developing approaches for modulation of the microbiota.^1^ While much research has centered on the gut microbiome, the microbiome in the respiratory tract has gained increasing attention, with growing evidence linking its dysbiosis to lung diseases such as asthma, chronic obstructive pulmonary disease (COPD), pulmonary fibrosis, pneumonia, acute lung injury, and lung cancer.^2^^,^^3^ Recent studies have demonstrated the roles of respiratory microbiota in responding to environmental exposure,^4^^,^^5^ and modulating lung immunity and inflammation.^6^^,^^7^

By transferring the microbial community from a donor to a recipient, microbiota transplantation has emerged as a promising approach for investigations into causality between microbiota and diseases, as well as the development of microbiota-based therapies. Fecal microbiota transplantation (FMT) is now a routine approach in pre-clinical studies to establish causality between microbiota and disease. Clinically, FMT has been approved for treating conditions such as *Clostridioides difficile* infection.^8^^,^^9^ Microbiota transplantation has also been explored in other niches such as the vagina.^10^^,^^11^ However, approaches in manipulating the respiratory microbiota remain largely understudied. Previous studies from us and others have attempted to modulate the respiratory microbiome in murine models through the direct administration of specific bacterial taxa via intranasal or intratracheal inoculation.^6^^,^^7^^,^^12^, ^13^, ^14^ These interventions have demonstrated immunomodulatory effects, confirming that targeted microbial supplementation can influence host immunity in the respiratory tract. A more translatable approach was developed by Nicola et al,^15^ who engineered an inhaled live biotherapeutic using active *Lactobacillus* strains, showing efficacy in modifying lung pathogenesis in bronchopulmonary dysplasia and COPD. Respiratory microbiota transplantation (RMT) has also been occasionally used in animal models, to establish causal roles of the microbiome in disease pathogenesis.^16^^,^^17^ Despite these progresses, an optimized, standardized protocol for RMT remains lacking. The development of RMT is impeded by challenges including the technical hurdle in establishing stable engraftment, as well as a critical knowledge gap regarding which experimental parameters most critically determine successful outcomes. Moreover, whether and how RMT impacts host metabolic and immunological characteristics remains unknown.

In this study, we developed an optimized RMT approach in murine model, using donor respiratory samples from both murine and human sources. We systematically evaluated the combined effects of storage conditions, delivery routes, and dosing regimens on RMT outcomes. We further investigated whether RMT reshapes metabolic and immunological characteristics in recipient mice. Our goal is to provide a standardized framework for modulating the respiratory microbiome in preclinical models, to assess the impact of RMT on host metabolomic and immunological characteristics, and to facilitate its clinical application.

## Methods

### Specific pathogen-free (SPF)-to-germ-free (GF) mouse microbiota transplantation

All animal experiments were approved by the Institutional Animal Care and Use Committee of South China Normal University at Guangzhou, China (ethical approval reference No. SCNU-SLS-2022-012). For all animal experiments in this study, 6- to 8-week-old male C57BL/6 mice were used. GF mice were housed in the First Affiliated Hospital of Sun Yat-Sen University (Guangzhou, China). Bronchoalveolar lavage fluid (BALF) was collected from 6- to 8-week-old SPF mice. Three sequential lavages were performed per mouse, yielding approximately 2 mL per mouse. A total of 20 SPF mice were used for microbiota transplantation to GF mice. The BALF samples of these SPF mice were pooled and centrifuged at 2500 × *g* for 7 min to pellet host cells. Culture experiments suggest modest loss of bacteria (∼9.63%, 133.3/1383.3 colony-forming unit [CFU]/mL) during this centrifugation step. The supernatant was carefully retained, and was centrifuged at 10,000 × *g* for 10 min to pellet bacterial cells. The bacterial pellet was divided into two portions: one portion was mixed with an equal volume of 20% glycerol and stored at −80°C until microbiota transplantation, while the other portion was processed in fresh. The pellet was resuspended in sterile phosphate-buffered saline (PBS) to achieve a resuspended bacterial suspension with an absorbance at 600 nm (*A*_600_) of 0.5. For transplantation, 50 μL of the suspension was administrated to each mice each time. The transplantation was conducted either daily for 7 days or every other day for 14 days. The combination of sample storage condition (fresh versus frozen), administration route (intranasal versus intratracheal), and duration (7 days or 14 days) generated eight treatment groups. BALF was collected from the recipient mice as described above. The lung lobes of the recipient mice were collected and transferred to a homogenizer containing 500–800 µL of physiological saline and homogenized by a FastPrep–24^TM^ (MP Biomedicals, Santa Ana, CA, USA) at 5.0 m/s for 20 s in 2-mL screw-cap tubes containing a mixture of 1.0 mm and 4.0 mm zirconia beads. BALF and lung samples of all recipient mice, along with the remaining BALF samples of donor SPF mice, were stored at −80°C for 16S ribosomal RNA (rRNA) gene sequencing. Quantitative polymerase chain reaction (PCR) was performed on a 7500 Real-Time PCR System (Applied Biosystems, Foster City, California, USA) using primers for bacterial 16S rRNA gene (F: GTGSTGCAYGGYTGTCGTCA, R: ACGTCRTCCMCACCTTCCTC). A detailed protocol for the respiratory microbiota transplantation (RMT), as implemented in this study, is provided in Appendix 1.

### Cecal ligation and puncture (CLP)-to-SPF mouse microbiota transplantation

The CLP procedure was performed as described previously.^18^^,^^19^ Briefly, mice were anesthetized and the cecum was exposed through a 1–2 cm midline laparotomy under aseptic conditions. The distal half of cecum was ligated, and a single through-and-through puncture was made using an 18 G needle, allowing a small amount of fecal material to be extruded from the perforated site. The peritoneum was then closed and the mice were resuscitated with a subcutaneous administration of 1 mL sterile saline. A total of 30 CLP mice were used for microbiota transplantation to SPF mice. BALF samples of CLP mice were collected, pooled, and processed, and transplanted to SPF mice under the same conditions as described above. Before RMT, SPF recipient mice received intranasal antibiotic pretreatment for 7 days to clear the endogenous respiratory microbiota, as described previously.^6^ BALF and lung tissue samples of all recipient mice, along with the BALF samples of donor CLP mice, were stored at −80°C for 16S rRNA gene sequencing.

### Human-to-mouse microbiota transplantation

BALF sample (∼30 mL) was collected from a 61-year-old male patient with COPD (body mass index 22.5 kg/m^2^, forced expiratory volume in 1 second/forced vital capacity [FEV_1_/FVC] 59.1%) and induced sputum sample (∼0.8 g) from a 58-year-old male patient with COPD (body mass index 27.6 kg/m^2^, FEV_1_/FVC 27.5%) at the First Affiliated Hospital of Guangzhou Medical University. The samples were processed as described previously.^6^ The protocol was approved by the ethics committee of the First Affiliated Hospital of Guangzhou Medical University (No. 2017-22). Both patients provided written informed consent. BALF was collected via clinical bronchoscopy. During bronchoscope insertion, suction was avoided to prevent contamination from the upper respiratory tract microbiota. The bronchoscope was wedged into the right middle lobe or lingula, and bronchoalveolar lavage was performed using sterile physiological saline. The patient underwent 3–4 lavage cycles, yielding approximately 30 mL of BALF, from which approximately 4 mL was aliquoted as one donor sample for transplantation. The collected BALF was kept on ice and centrifuged at 2500 × *g* and 4 °C for 7 min to pellet host cells. The supernatant was retained and centrifuged at 10,000 × *g* and 4°C for 10 min to obtain a bacterial pellet. For sputum samples, an equal volume of N-acetylcysteine was added to liquefy the sample after collection. The resulting supernatant was retained, while the pellet was discarded. The supernatant was subsequently centrifuged at 10,000 × *g* and 4°C for 10 min to obtain a bacterial pellet. The bacterial pellet was mixed with an equal volume of 20% glycerol and stored at −80°C until microbiota transplantation. During transplantation, the cryopreserved samples were thawed on ice, centrifuged at 10,000 × *g* and 4°C for 10 min, and the glycerol supernatant was removed to retain the bacterial pellet. The pellet was then resuspended in sterile PBS to achieve a resuspended bacterial suspension with an *A*_600_ of 0.5. For transplantation, 50 μL of the suspension was administrated to each mice each time. Human-to-mouse microbiota transplantation was conducted every other day for 14 days, either intranasally or intratracheally. BALF and lung tissue samples of the recipient mice, along with the human BALF and sputum samples as donors, were stored at −80°C for 16S rRNA gene sequencing.

### Microbiota sequencing and data analyses

Bacterial genomic DNA was extracted from the samples collected above using a Total Nucleic Acid Extraction Kit (Bioeasy Technology, Inc., Shenzhen, China) as per the manufacturer’s instructions. Reagent controls for DNA extraction (*n* = 1) and PCR (*n* = 2) were included and sequenced along with the test samples (Supplementary Table 1). The V4 hypervariable region of bacterial 16S rRNA gene was PCR amplified using primers 515F (5-GTGCCAGCMGCCGCGGTAA-3) and 806R (5-GGACTACHVGGGTWTCTAAT-3) and the amplicons were sequenced using Illumina NovaSeq platform (Illumina, Inc., San Diego, California, USA). Sequencing reads were processed using QIIME 2.0 pipeline (v2.0, https://qiime2.org).^20^ Reads were quality filtered and denoised using DADA2 as implemented in QIIME 2.0 to generate amplicon sequence variants (ASVs).^21^ The taxonomy of each ASV was assigned using a Naive Bayes classifier implemented in QIIME 2.0 pre-built using the 515F–806R region of 16S rRNA gene sequences for all 99% operation taxonomic units in Greengene database. Rarefaction was performed according to the lowest sequencing depth of all samples.

### Metabolomic and transcriptomic analyses

Mouse BALF supernatants were subjected to non-targeted metabolomics using a Xevo G2-XS QTof (Waters Corporation, Milford, Massachusetts, USA). Raw metabolome data were converted into mzXML format. Ion features were extracted using Progenesis QI (v2.2, Waters Corporation, Milford, Massachusetts, USA). Low-quality ions (missing in over 50% of quality control samples or over 80% of test samples), or ions with relative standard deviation >30%, were filtered from downstream analysis. Metabolites were identified by searching against Human Metabolome Database (HMDB) (v5.0, University of Alberta, Edmonton, Alberta, Canada; https://hmdb.ca),^22^ Metabolite Link (METLIN) (v3.7.1, Scripps Research, La Jolla, California, USA; https://metlin.scripps.edu)^23^ and Kyoto Encyclopedia of Genes and Genomes (KEGG v96.0, Kanehisa Laboratories, Kyoto, Japan; https://www.kegg.jp).^24^ Annotation of the origin of metabolites (host or microbiota) was conducted using MetOrigin (v2.0, https://metorigin.met-bioinformatics.cn).^25^

RNA was extracted from mouse BALF cells using Qiagen RNease Mini kit (Qiagen, Hilden, Germany) for messenger RNA (mRNA)-sequencing using the Illumina NovaSeq platform. The raw reads for host transcriptome were quality filtered using Cutadapt (v2.5, https://cutadapt.readthedocs.io)^26^ and aligned to mouse genome Genome Reference Consortium MouseBuild 39 (GRCm39) using Hisat 2 (v2.1.0, http://daehwankimlab.github.io/hisat2/).^27^ The gene count matrix was obtained using RNA-Seq by Expectation-Maximization (RSEM) (v1.3.3, https://deweylab.github.io/RSEM).^28^

### Statistical analyses

Bray-Curtis dissimilarity index was used to assess the similarity in microbiome composition between donor and recipient mice. To assess the contribution of individual microbial taxa to donor–recipient microbiota similarity, a leave-one-out analysis was performed, by recalculating the Bray-Curtis dissimilarity of donor and recipient samples with one genus excluded at a time, as described previously.^29^ The contribution of that species to the donor–recipient similarity was defined as the deviation of donor–recipient Bray–Curtis dissimilarity index from its original value when that genus was excluded (BC_taxon excluded_ – BC_original_).

For metabolomic data, trend analysis was performed using fuzzy c-means algorithm with timeclust function of TCseq package (v3.18, https://bioconductor.org/packages/TCseq/).^30^ Briefly, a pseudo-time-course table was constructed by calculating fold changes of the metabolites in the GF (control), glycerol-preserved intranasal 14-day (GN14D) and glycerol-preserved intratracheal 14-day (GT14D) groups relative to the SPF group, with a minimum fold change threshold of 1.5. Subsequently, fuzzy c-means clustering was performed on the data using Euclidean distance measure with standardized expression profiles (mean-centered and unit-variance scaled for each metabolite). The optimal number of clusters was determined through evaluation of clustering stability and biological interpretability. Specifically, we focused on co-abundance metabolite clusters showing enrichment or depletion in GF versus SPF mice, which were reversed in microbiota transplanted mice.

For transcriptomic data, trend analysis was performed using fuzzy c-means algorithm with timeclust function of TCseq package with default parameters (v3.18, https://bioconductor.org/packages/TCseq/),^30^ as described for the metabolomic data. Specifically, we focused on co-expressed genes showing enrichment or depletion in GF versus SPF mice, which were reversed in microbiota transplanted mice. Genes in the corresponding clusters were subjected to pathway enrichment analysis via MetaCore (Clarivate Analytics, London, UK) against MetaBase canonical pathways (v6.30.68780, Clarivate Analytics). To determine the statistical significance of enriched pathways, we applied a hypergeometric test. This analysis assessed whether the observed number of genes from a given pathway in a cluster significantly exceeded the expected number by chance, using the total number of annotated genes in the pathway (*n* = 13,135) as the background population.

For Bray-Curtis dissimilarity, data are presented as mean ± standard deviation (SD). For the relative abundance of bacterial taxa, data are presented as median and range. Paired Student’s *t*-test was used to compare the bacterial viability between fresh and glycerol-frozen samples. For comparisons of Bray-Curtis dissimilarity and relative abundances of microbiome genera among different transplantation conditions, the Wilcoxon rank-sum test was employed. For comparison of microbiome genera between the transplantation recipients and their donors, a one-sample Wilcoxon signed-rank test was used to test whether the change in abundance of a genus in the recipient samples compared to the corresponding donor significantly deviated from zero. All statistical analyses and Bray-Curtis dissimilarity index calculations were performed using R software (version 4.3.1, R Core Team, Vienna, Austria), utilizing the “vegan” package for dissimilarity index and the “stats” package for non-parametric tests. False discovery rate (FDR) correction was implemented to control for multiple testing where applicable. A *P*–value (adjusted where applicable) < 0.05 was considered statistically significant.

## Results

### GF mice-based RMT

**Fig. 1:**
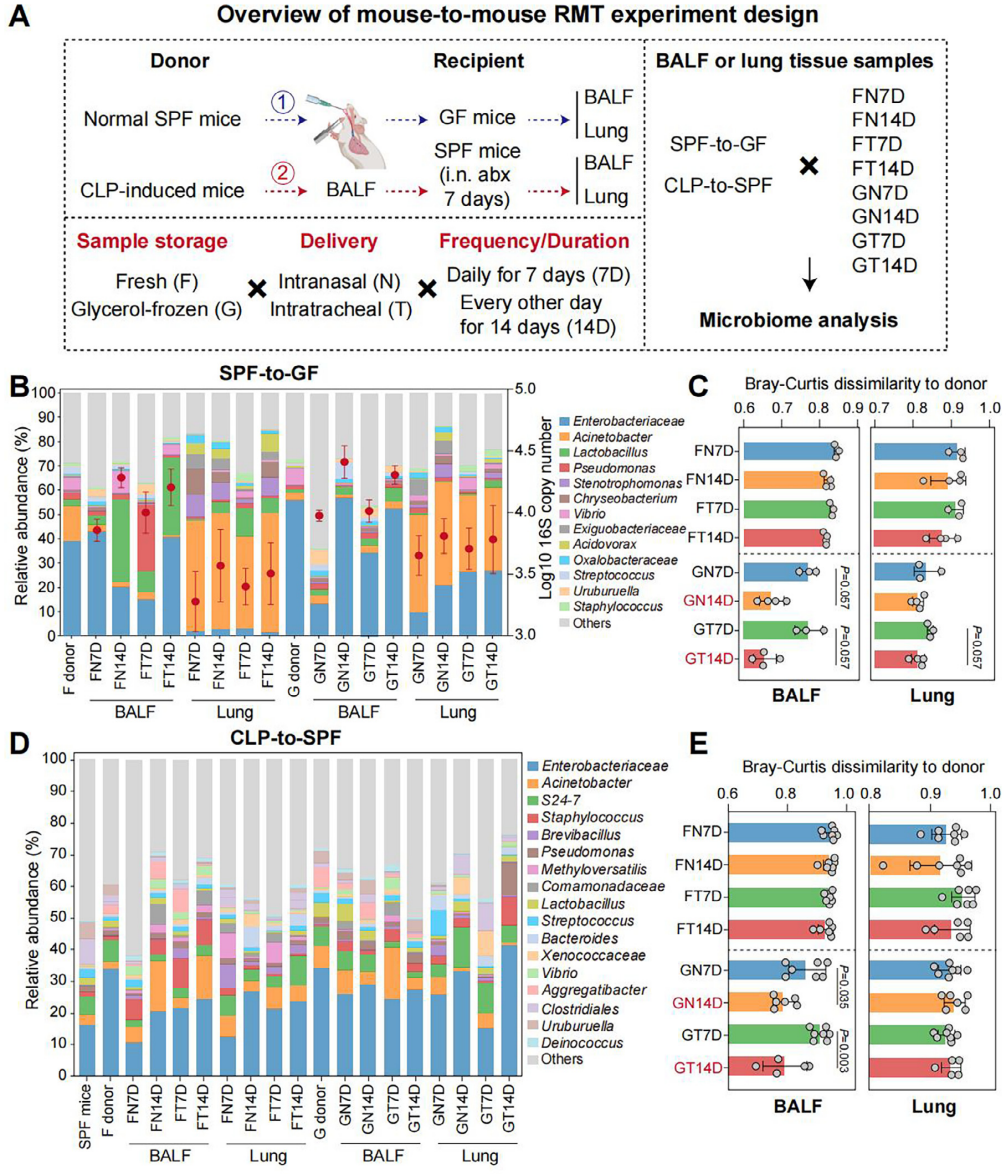
Mouse-to-mouse RMT. (A) Overview of mouse-to-mouse RMT design. (B–C) Results for RMT from SPF to GF mice. (B) Relative abundance of major genus-level taxa for fresh-processed (F donor) and glycerol-preserved BALF samples of the donor mice (G donor), as well as BALF and lung samples of the recipient mice, where microbiota was transplanted intranasally or intratracheally, every day for 7 days or every other day for 14 days. Also shown are the log_10_ copy numbers of 16S rRNA gene indicating total bacterial load of BALF and lung samples of the recipient mice in each group (mean ± SD). (C) Bray-Curtis dissimilarity index calculated from lung and BALF samples of recipient mice and their corresponding donors in each microbiota transplantation group (*n* = 3–4 per group, mean ± SD). (D–E) Results for RMT from CLP-induced SPF mice to normal SPF mice. (D) Relative abundance of major genus-level taxa for fresh-processed (F donor) and glycerol-preserved BALF samples of CLP mice as donors (G donor), BALF samples of normal SPF mice (*n* = 2), and BALF and lung samples of the microbiota recipient mice. (E) Bray-Curtis dissimilarity between recipient and donor samples in each microbiota transplantation group, assessed in lung tissue and BALF separately (*n* = 5–7 per group, mean ± SD). BALF, Bronchoalveolar lavage fluid; CLP, Cecal ligation and puncture; GF, Germ-free mice; i.n. abx, Intranasal and antibiotics; RMT, Respiratory microbiota transplantation; rRNA, Ribosomal RNA; SD, Standard deviation; SPF, Specific pathogen-free mice.

To explore the feasibility of RMT, we first designed a mouse-to-mouse transplantation experiment using SPF mice as donors and GF mice as recipients. Microbial cells were isolated from BALF samples of donor mice (pooled, *n* = 20). Following transplantation, we collected BALF and lung tissue samples from recipient mice for comparative microbiome analysis against donor samples to evaluate transplantation efficiency (Fig. 1A). According to Bray-Curtis dissimilarity indices, BALF microbiota of recipient mice showed an overall higher similarity to donor BALF microbiota than did the recipient lung tissue microbiota (Bray-Curtis dissimilarity: BALF = 0.769 ± 0.074, Lung = 0.861 ± 0.046, Wilcoxon rank-sum test, *W* = 135.0, *P* = 1.04e-5), with a disproportionate over-representation of *Acinetobacter* in the lung tissue (Fig. 1B). Therefore, we focused subsequent comparisons on the recipient BALF samples. Comparison of fresh and glycerol-frozen samples revealed a generally greater donor–recipient microbiota similarity in the glycerol-frozen group **(**Bray-Curtis dissimilarity: glycerol = 0.710 ± 0.061, fresh = 0.829 ± 0.013, Wilcoxon rank-sum test, *W* = 194.0, *P* = 1.99e-7). A possible explanation for this observation is that fresh samples were maintained at 4°C during the entire 7 or 14-day period of transplantation, potentially compromising microbial viability. On the other hand, glycerol-frozen samples, being stored at −80°C, may have benefited from better preservation of microbial composition. Consistent with this finding, culture experiments confirmed superior preservation of bacterial viability in glycerol-frozen samples (total bacterial load: 1520.0 ± 567.5 CFU/mL) compared to those stored at 4°C (780.0 ± 228.0 CFU/mL) over a 14-day period (paired *t*-test, *t* = 2.369, *P* = 0.077). Therefore, the cryoprotective properties of glycerol appear to maintain microbiota integrity more effectively even than fresh samples during the extended time of transplantation.

Comparison of delivery conditions revealed a trend toward a greater donor–recipient microbiota similarity in BALF samples in the 14-day intermittent regimen compared to the 7-day daily administration, for both intranasal and intratracheal delivery routes, at the borderline of significance (Bray-Curtis dissimilarity: GN14D [glycerol-preserved samples, delivered intranasally every other day, for a duration of 14 days] = 0.674 ± 0.033, GN7D [glycerol-preserved samples, delivered intranasally every day for 7 days] = 0.770 ± 0.022, Wilcoxon rank-sum test, *W* = 12.0, *P* = 0.057; GT14D [glycerol-preserved samples, delivered intratracheally every other day, for a duration of 14 days] = 0.656 ± 0.030, GT7D [glycerol-preserved samples, delivered intratracheally every day for 7 days] = 0.772 ± 0.037, Wilcoxon rank-sum test, *W* = 12.0, *P* = 0.057, Fig. 1C), suggesting that intermittent administration better preserves microbial community likely by providing an extended time window for microbial colonization. No significant difference was observed between intranasal and intratracheal delivery routes. In support, quantitative PCR revealed a higher absolute total bacterial load in BALF samples of recipient mice subjected to 14-day intermittent regimen compared to 7-day daily administration, independent of sample storage condition or delivery route (Fig. 1B). Based on these results in GF mice, we conclude that RMT achieves optimal performance when using glycerol-frozen samples, administered either intranasally or intratracheally, intermittently over 14 days.

### SPF mice-based RMT

GF mice are ideal for evaluating microbiota transplantation efficiency, but their relatively high cost and limited availability restrict their utility in practice. To assess the generalizability of the results observed in GF mice, we also performed a mouse-to-mouse transplantation in SPF mice. To be able to distinguish the donor and recipient microbiota, we induced an endogenous dysbiosis of the respiratory microbiota by constructing a CLP model. CLP is an established procedure for establishing murine model of sepsis.^31^ By inducing a gut-derived polymicrobial infection to the abdominal cavity and other distal organs (i.e., lungs) mimicking human sepsis, this model is known to induce a trackable shift of the respiratory microbiota with enrichment of gut bacteria.^32^ Indeed, an enrichment of gut-derived taxa, notably *Enterobacteriaceae*, was observed in BALF of CLP-induced mice compared with conventional SPF mice (*n* = 2, Fig. 1D). The recipient SPF mice were subjected to intranasal administration of antibiotics (for 7 days) to effectively disrupt the respiratory microbiota,^6^ prior to RMT. The BALF of the CLP mice (pooled, *n* = 30) was then used as donors for RMT in a similar experimental setting to that used for the GF mice. Consistent with the findings in GF mice, a significantly closer resemblance of donor–recipient microbiota in BALF samples was also observed for the GN14D and GT14D groups compared with GN7D and GT7D groups (Bray-Curtis dissimilarity: GN14D = 0.787 ± 0.036, GN7D = 0.866 ± 0.061, Wilcoxon rank-sum test, *W* = 36.0, *P* = 0.035, GT14D = 0.791 ± 0.074, GT7D = 0.912 ± 0.026, Wilcoxon rank-sum test, *W* = 35.0, *P* = 0.003, Fig. 1E, Supplementary Fig. 1A), supporting RMT using glycerol-frozen samples in a 14 day-intermittent manner as the optimal procedure for mouse-to-mouse transplantation.

### Multi-omics analysis of GF mice receiving RMT

GF mice offer a unique opportunity for assessing the colonization capability of individual members of the microbiota in the lung, as well as their influence on the local metabolism and immunity. To estimate efficiency of colonization for each individual taxon, we assessed differences in genus-level taxa between the BALF of recipient GF mice and that of SPF donor. Among all 45 genera examined (relative abundance >0.001), 8 genera were significantly enriched in recipient samples compared to the corresponding donors (Wilcoxon signed-rank test, *P* < 0.05), while 21 genera were significantly depleted (Supplementary Table 2). The relative abundances of the remaining 16 genera did not differ significantly between donor and recipient samples. Thus, they were considered to maintain their relative abundance during transplantation, implying a state of stable colonization (Supplementary Fig. 1B). To assess the contribution of individual genera to donor–recipient microbiota similarity, we conducted a leave-one-out analysis by assessing the average difference in Bray-Curtis dissimilarity between donor and recipient samples in GN14D and GT14D groups (BC_taxon excluded_ - BC_original_), with each genus excluded iteratively. The 16 "stably colonized" genera in general contributed positively to donor–recipient microbiota similarity (Supplementary Fig. 1B, Supplementary Table 2). Of them, *Enterobacteriaceae, Stenotrophomonas*, and *Acetobacteraceae* exhibited the greatest contribution, suggesting they were likely key microbial factors driving the donor–recipient microbiota similarity.

**Fig. 2:**
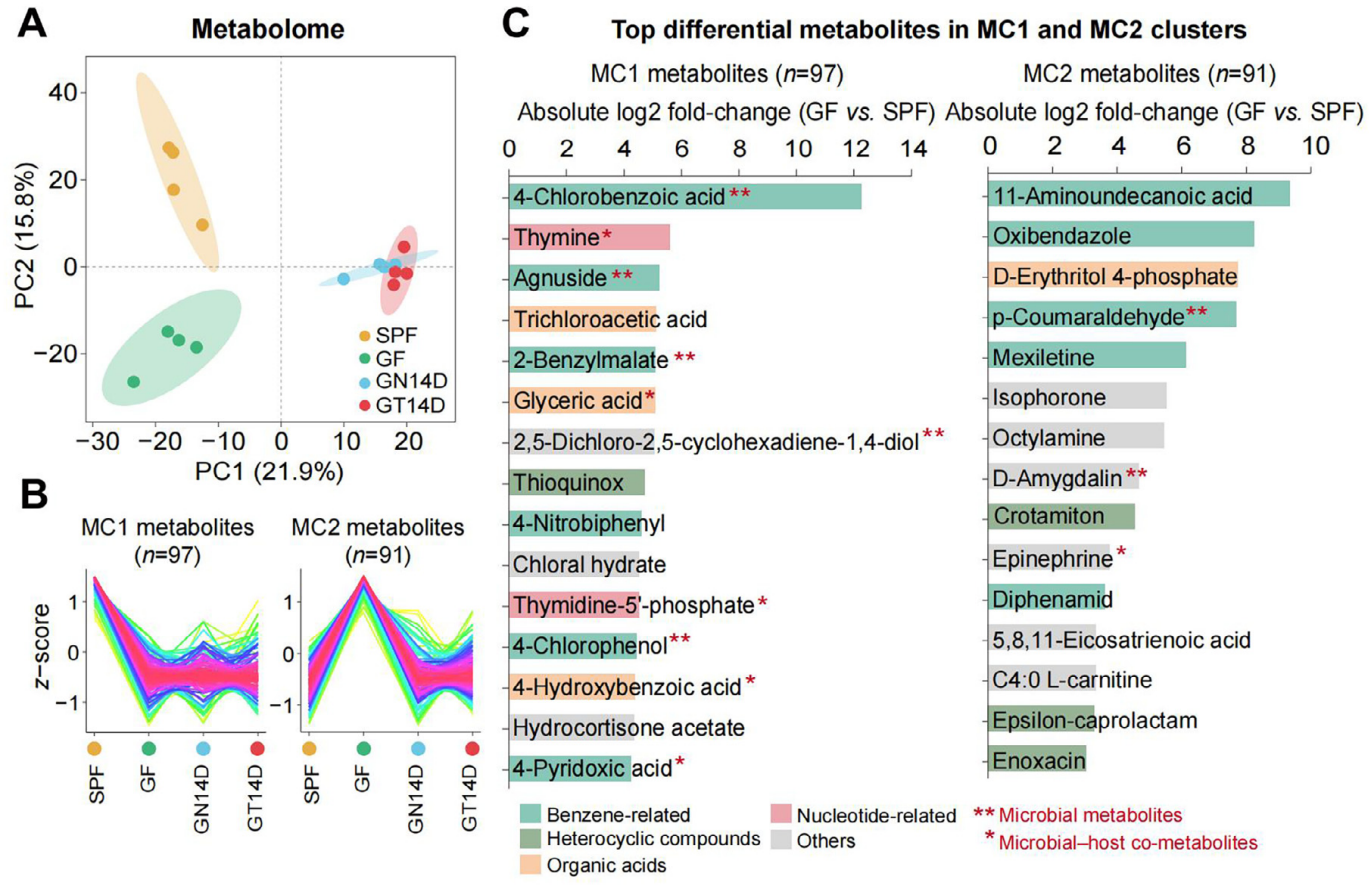
Metabolomic characterization of SPF, GF and respiratory microbiota transplanted mice. (A) Principal component analysis for lung metabolomics of SPF, GF and microbiota transplanted mice (GN14D and GT14D). (B) Clustering analysis revealed two clusters showing enrichment or depletion of metabolites in GF versus SPF mice that were reversed in microbiota transplanted mice. (C) The top 15 metabolites in MC1 and MC2 with highest absolute fold-changes between SPF and GF mice, colored by their chemical categories. The metabolites inferred to be putatively produced by microbiota are highlighted in double asterisks. The metabolites inferred to be co-produced by microbiota and host are highlighted in single asterisk. GF, Germ-free mice; GN14D, Glycerol-preserved samples, delivered intranasally every other day, for a duration of 14 days; GT14D, Glycerol-preserved samples, delivered intratracheally every other day, for a duration of 14 days; MC1, Metabolomic cluster 1; MC2, Metabolomic cluster 2; PC1, Principal component 1; PC2, Principal component 2; SPF, Specific pathogen-free mice.

To determine whether microbiota depletion influences lung metabolism and host gene expression, and if these effects are reversible by transplantation, we compared the lung metabolome and host transcriptome across SPF, GF, and GF mice that had received RMT (GN14D and GT14D groups). Marked differences in lung metabolome were observed in GF mice compared to SPF controls, while microbiota transplantation partially attenuated these differences (Fig. 2A). The high similarity between the GT14D and GN14D groups in metabolome further indicates comparable transplantation efficiency using both methods (Fig. 2A). We performed a trend analysis to identify metabolites that were differentially abundant between GF and SPF mice and whose differences were partially attenuated by microbiota transplantation. Among all 992 metabolites with available annotation, 97 and 91 metabolites were depleted and enriched in GF mice, respectively, trends that were reversed following microbiota transplantation (Fig. 2B, Supplementary Table 3). Of note, when ranking the 97 metabolites by their fold-changes in GF versus SPF mice, 10 of 15 top metabolites were inferred to be capable of production by microbiota (using MetOrigin 2.0^20^), and five metabolites, including 4-chlorobenzoic acid, agnuside, 2-benzylmalate, were predicted to be putatively produced by microbiota (Fig. 2C).

**Fig. 3:**
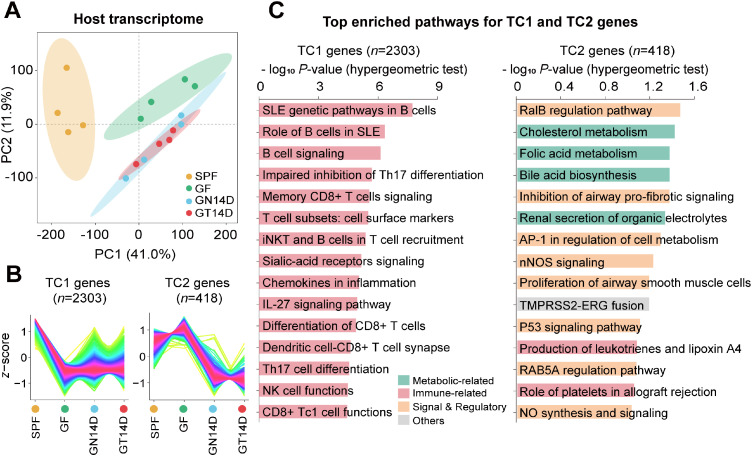
Host transcriptomic characterization of SPF, GF and respiratory microbiota transplanted mice. (A) Principal component analysis for host transcriptomics of SPF, GF and microbiota transplanted mice (GN14D and GT14D). (B) Clustering analysis revealed two clusters showing upregulation or downregulation of host genes in GF versus SPF mice that were reversed in microbiota transplanted mice. (C) The top 15 most significantly enriched pathways (based on hypergeometric test) for genes in TC1 and TC2, colored by their functional categories. AP-1, Activator protein 1; CD8, Cluster of differentiation 8; ERG, ETS-related gene; GF, Germ-free mice; GN14D, Glycerol-preserved samples, delivered intranasally every other day, for a duration of 14 days; GT14D, Glycerol-preserved samples, delivered intratracheally every other day, for a duration of 14 days; IL-27, Interleukin 27; iNKT, Invariant natural killer T cells; NK, Natural killer; nNOS, Neuronal nitric oxide synthase; NO, Nitric oxide; P53, Tumor protein p53; PC1, Principal component 1; PC2, Principal component 2; RAB5A, Ras-related protein Rab-5A; RalB, Ras-related protein Ral-B; SLE, Systemic lupus erythematosus; SPF, Specific pathogen-free mice; TC1, Transcriptomic cluster 1; TC2, Transcriptomic cluster 2; Tc1 cell: Type 1 CD8+ cytotoxic T cell; Th17, T helper 17 cells; TMPRSS2, Transmembrane serine protease 2.

Transcriptomic analysis revealed an overall pattern similar to the metabolomic data. Specifically, gene expression profiles differed markedly between GF and SPF mice. Microbiota transplantation (GT14D and GN14D) in GF recipients shifted their transcriptomic profiles toward those of SPF mice (Fig. 3A). Trend analysis revealed 2303 and 418 genes that were downregulated and upregulated in GF versus SPF mice, and were then reversed in the transplanted groups (Fig. 3B). Pathway analysis revealed that the downregulated genes were predominantly involved in immune responses, including B cell and T cell signaling, natural killer (NK) cell functions, and interleukin 27 (IL-27) signaling (Fig. 3C, Supplementary Table 4), suggesting an impaired immune status in GF mice that was partially rescued by microbiota transplantation. Conversely, the upregulated genes were enriched for pathways related to a diverse range of host metabolic, signaling, and regulatory processes (Fig. 3C, Supplementary Table 4).

### Human-to-mouse RMT

**Fig. 4:**
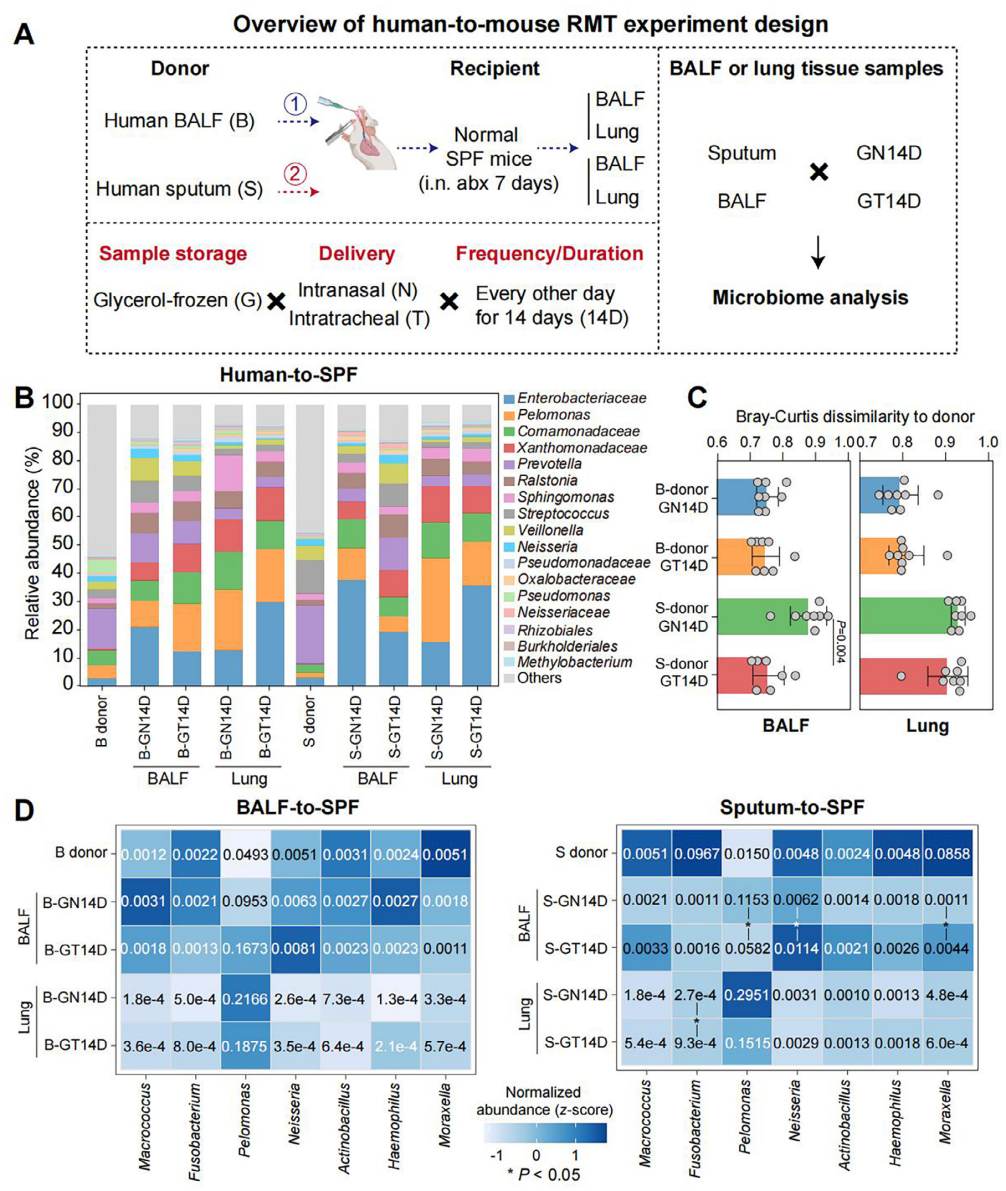
Human-to-mouse RMT. (A) Overview of human-to-mouse RMT design. (B–C) Results for RMT from human sputum or BALF samples to SPF mice. (B) Relative abundance of major genus-level taxa for glycerol-preserved (G) BALF (B donor) and sputum samples (S donor), as well as BALF and lung samples of the recipient mice, where microbiota was transplanted intranasally (N) or intratracheally (T), every other day for 14 days (14D). (C) Bray-Curtis dissimilarity index between recipient lung or BALF samples and the corresponding donor BALF or sputum samples in each microbiota transplantation group (*n* = 7–8 per group, mean ± SD). (D) Comparison of relative abundance of seven bacterial genera more abundant in human samples between donor (B donor and S donor) and recipient (mouse lung and BALF) samples in GN14D and GT14D groups (medians are shown). Genera significantly different in abundance between GN14D and GT14D are indicated in asterisks (FDR <0.05). BALF, Bronchoalveolar lavage fluid; FDR, False discovery rate; GN14D, Glycerol-preserved samples, delivered intranasally every other day, for a duration of 14 days; GT14D, Glycerol-preserved samples, delivered intratracheally every other day, for a duration of 14 days; i.n. abx, Intranasal and antibiotics RMT, Respiratory microbiota transplantation; SD, Standard deviation; SPF, Specific pathogen-free.

Having established optimal conditions for mouse-to-mouse RMT, we next applied the GN14D and GT14D regimens to evaluate the feasibility of human-to-mouse RMT using human BALF or sputum as donor samples (Fig. 4A). One BALF sample and one sputum sample were collected from two different COPD patients. Microbial cells from BALF and sputum samples were processed, glycerol-frozen, and administered intranasally or intratracheally to antibiotic-pretreated SPF mice over a 14-day intermittent schedule. Similar to mouse-to-mouse RMT, recipient microbiota more closely resembled the donor when assessed in BALF than in lung tissue (Fig. 4B–C). For human BALF donors, transplantation efficiency was comparable between GN14D and GT14D (Bray-Curtis dissimilarity: GN14D = 0.754 ± 0.331, GT14D = 0.749 ± 0.042, Wilcoxon rank-sum test, *W* = 38.0, *P* = 0.574). However, for sputum donors, GT14D resulted in significantly greater donor resemblance than GN14D (Bray-Curtis dissimilarity: GN14D = 0.879 ± 0.056, GT14D = 0.757 ± 0.048, Wilcoxon rank-sum test, *W* = 53.5, *P* = 0.004), suggesting intratracheal administration could be superior for sputum-based transplantation, likely by enabling a better lung colonization of the sputum microbiota (Fig. 4B–C, Supplementary Fig. 1A).

To assess transplantation efficiency of individual taxa, we tracked seven bacterial genera that were reasonably abundant in human BALF and sputum samples (relative abundance >1e-3 in BALF and sputum donors), but relatively rare in BALF of recipient SPF mice (relative abundance <1e-4 in normal SPF mice). For transplantation using BALF donor samples, *Macrococcus* (relative abundance: donor 0.0012, recipient 0.0021 [0.0002–0.0058], Wilcoxon signed-rank test *V* = 115, *P* = 0.016), *Pelomonas* (donor 0.0493, recipient 0.0945 [0.0242–0.4460], *V* = 126, *P* = 0.001), and *Neisseria* (donor 0.0051, recipient 0.0081 [0.0034–0.0150], *V* = 105, *P* = 0.011) were significantly higher in the recipient BALF samples compared to donor samples; *Actinobacillus* (donor 0.0031, recipient 0.0023 [0.0007–0.0044], *V* = 13.5, *P* = 0.027) and *Moraxella* (donor 0.0051, recipient 0.0015 [0.0002–0.0036], *V* = 0, *P* < 0.001) were significantly lower, and *Fusobacterium* and *Haemophilus* were not significantly different (Supplementay Fig. 2). For transplantation using sputum samples, *Neisseria* (donor 0.0048, recipient 0.0089 [0.0031–0.0143], *V* = 111.0, *P* = 0.002) and *Pelomonas* (donor 0.0150, recipient 0.0836 [0.0157–0.2289], *V* = 120.0, *P* = 6.1e-5) were significantly higher in the recipient compared to donor samples, and all other species were significantly lower (*P* < 0.05, Supplementary Fig. 2). These results suggest that *Pelomonas* and *Neisseria* were more likely to colonize and proliferate in the murine lung, while the other species, in particular *Actinobacillus* and *Moraxella*, were less likely to establish a stable colonization. The abundances of the seven genera were comparable between GN14D and GT14D when using BALF donors (Fig. 4D). In comparison, sputum-based transplantation via GT14D resulted in a higher relative abundance for five genera in recipient BALF samples than that via GN14D, reaching a level more closely matching that in the original donor sample (Fig. 4D). This enrichment was particularly notable for *Neisseria* (GN14D 0.0062 [0.0031–0.0143], GT14D 0.0114 [0.0065–0.0128], Wilcoxon signed-rank test, *W* = 9.0, *P* = 0.029) and *Moraxella* (GN14D 0.0011 [0.0005–0.0017], GT14D 0.0044 [0.0007–0.0109], Wilcoxon signed-rank test, *W* = 7.5, *P* = 0.020). These results support that the GT14D regimen achieves higher transplantation efficiency for major microbial taxa in human sputum samples.

## Discussion

The RMT approach, as developed in this study, could have implications in lung microbiota translational research. Essentially, it provides a tool to directly test whether a dysbiotic lung microbiota from a diseased host (either mouse or human) can induce pathology in a healthy recipient, or conversely, if a healthy lung microbiota can confer resilience or promote recovery from the dysbiotic state, thereby moving beyond correlation to causality between specific respiratory microbial communities and health outcomes. Furthermore, this approach allows for further mechanistic studies of how individual members of the respiratory microbiota influences local immunity and metabolism,^14^^,^^33^ as demonstrated by our multi-omic characterization of SPF, GF and microbiota transplanted mice. From a therapeutic standpoint, this work lays the groundwork for developing RMT as a potential clinical intervention. By demonstrating engraftment of microorganisms from human donors to recipient lungs, our study provides a proof-of-concept for potentially treating refractory lung diseases, such as severe asthma, COPD, or fibrotic lung diseases, by resetting the respiratory microbiome with a healthy, donor-derived community.^34^

Despite these promises, our study has several limitations. First, although we evaluated key parameters such as sample storage, delivery route, and administration frequency, our testing was not exhaustive. For instance, in addition to intranasal and intratracheal delivery, nebulization may represent a promising alternative approach for RMT, as it enables noninvasive, clinically translatable delivery of microorganisms to the lower airways. However, a major limitation of nebulization is the difficulty in precisely controlling and quantifying the effective microbial dose deposited in the lungs, due to high inter-individual variability in inhalation patterns. This inherent variability complicates reliable assessment of transplantation efficiency and was the primary reason we did not include nebulization in the current study. Future studies could address this limitation by adopting standardized nebulization devices and advanced biomaterial-assisted strategies, such as hydrogel-based encapsulation systems.^35^ These approaches could enhance both the standardization of delivery and the preservation of microbial viability. Second, while our current 16S rRNA gene sequencing-based analysis provides robust assessment of microbiota compatibility at the overall community and genus levels, future studies using metagenomic sequencing will be essential to achieve species-resolution profiling and to elucidate the functional genomic basis of the observed transplantation effects. Third, although we identified specific bacterial taxa that stably colonized GF mouse lungs and may influence host metabolism and immunity, the mechanistic roles of individual microbes remain uncharacterized. Subsequent studies using gnotobiotic models colonized with defined microbial communities will be necessary to elucidate the causal role of key taxa in the observed metabolic and immunological changes. Fourth, for GF mice, the limited sample size (*n* = 3–4 per group) may have influenced statistical power, and the differences between GN7D and GN14D, as well as between GT7D and GT14D, were only at the borderline of significance. However, the same comparison in SPF mice, with larger group sizes (*n* = 5–7), reached statistical significance, supporting the superiority of the 14-day over the 7-day regimen. Finally, due to the invasive nature of sampling the murine respiratory microbiota, we were unable to perform longitudinal monitoring of the lung microbial dynamics in individual mice. Thus, the long-term stability of the transplanted microbiota and the durability of its functional effects on the host are key questions that warrant further exploration. In addition, future studies should incorporate background profiling of the microbiome in non-transplanted mice, to allow a more precise assessment of transplantation-induced microbiota changes.

In summary, we developed and optimized a robust protocol for RMT in mice, using donor samples from both murine and human sources. Our findings establish that a regimen of glycerol-preserved samples administered intermittently over 14 days yields optimal engraftment for mouse-to-mouse RMT, with both intranasal (GN14D) and intratracheal (GT14D) routes being effective. For human-to-mouse RMT, while GN14D and GT14D achieved comparable efficiency for transplanting microbiota from human BALF samples, GT14D is superior for sputum samples. We anticipate that the RMT approach will serve as a reference to empower future preclinical studies into the causal mechanisms of the respiratory microbiome. Furthermore, it establishes an essential proof-of-concept platform for translating RMT into a novel therapeutic strategy for respiratory diseases.

## Funding

This work was supported by National Key Research & Development Program of China (No. 2022YFA1304300), National Natural Science Foundation of China (Nos. 82495200, 82495201, 32170109), Major Project of Guangzhou National Laboratory (No. GZNL2023A02001), the Noncommunicable Chronic Diseases-National Science and Technology Major Project (No. 2024ZD0528400), and the Open Research Projects of the State Key Laboratory of Respiratory Diseases (No. SKLRD-Z-202507).

## Data availability

All raw sequencing and proteome data have been deposited in the Genome Sequence Archive in BIG Data Center (https://bigd.big.ac.cn), Beijing Institute of Genomics (BIG), Chinese Academy of Sciences. The raw 16S rRNA gene sequencing data are under PRJCA045652 (https://ngdc.cncb.ac.cn/bioproject/browse/PRJCA045652). The raw metabolomic data are under PRJCA045655 (https://ngdc.cncb.ac.cn/bioproject/browse/PRJCA045655). The raw host transcriptomic data are under PRJCA045653 (https://ngdc.cncb.ac.cn/bioproject/browse/PRJCA045653).

## CRediT authorship contribution statement

**Shifen Xu:** Investigation. **Xing Zhang:** Investigation. **Yunfeng Shi:** Investigation. **Xiang Tan:** Investigation. **Hanqin Cai:** Investigation. **Wei Cheng:** Investigation. **Lei Yang:** Investigation. **Xinzhu Yi:** Investigation. **Zhiming Xiang:** Supervision, Investigation. **Chao Cao:** Supervision, Investigation. **Hong Wei:** Supervision, Investigation. **Zhang Wang:** Writing – review & editing, Writing – original draft, Supervision, Investigation, Funding acquisition, Conceptualization.

## Declaration of competing interest

The authors declare that they have no known competing financial interests or personal relationships that could have appeared to influence the work reported in this paper.

## Appendix Supplementary materials

Image, application 1

Image, application 2
