# Supplementary material for: Respiratory microbiota transplantation: optimized framework and its impact on metabolic and immune characteristics
Source: Chin Med J Pulm Crit Care Med. 2026 Jun 8;4(2):184–92. doi: 10.1016/j.pccm.2026.05.002 (PMC13323557; doi:10.1016/j.pccm.2026.05.002)
Supplement: Supplementary file 1 [file mmc1.docx]

**
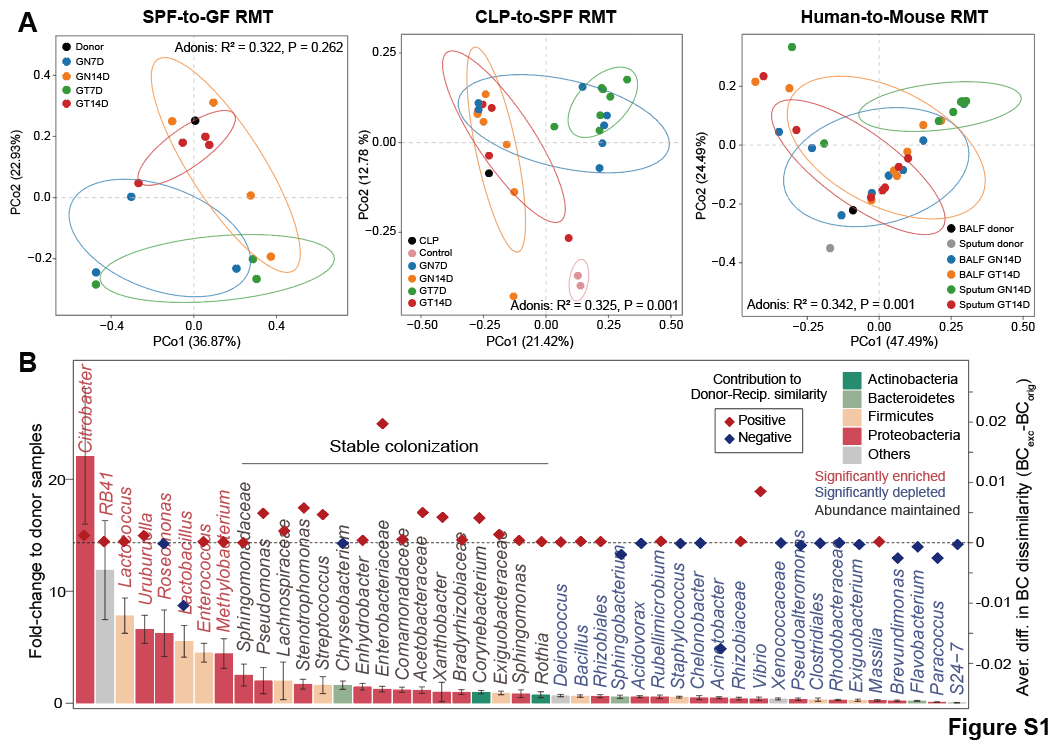
**

**Supplementary Fig. 1. (A)** Principal coordinate analysis (PCoA) plots for (1) specific pathogen-free (SPF)-to-germ-free (GF) mouse microbiota transplantation (donor, GN7D, GN14D, GT7D, GT14D), (2) cecal ligation puncture (CLP)-to-SPF mouse microbiota transplantation (CLP, control, GN7D, GN14D, GT7D, GT14D), and (3) human-to-mouse microbiota transplantation (bronchoalveolar fluid (BALF) donor, sputum donor, BALF GN14D, BALF GT14D, sputum GN14D, sputum GT14D). The PCoA results are overall consistent with the results based on the comparison of Bray-Curtis dissimilarity index. Specifically, for mouse-to-mouse transplantation, there was an overall closer resemblance of the recipient microbiota with donors for GN14D and GT14D, compared with GN7D and GT7D. For human-to-mouse transplantation, there was a closer resemblance of the recipient microbiota with sputum donor for GT14D than GN14D. (**B)** Barplot (mean±SD) showing fold-change of relative abundance of genus-level taxa (relative abundance >0.001) in recipient GF mice compared to corresponding donor samples. Genera significantly enriched and depleted in recipient compared to donor mice (Wilcoxon, *P*<0.05) are designated in red and blue, respectively. Genera that were not significantly different between donor and recipient mice are in black, implying a state of stable colonization. The contribution of each genus to the donor-recipient microbiota similarity is indicated as the average difference in Bray-Curtis dissimilarity between donor and recipient samples in GN14D and GT14D groups, when that genus was excluded (BC_taxon excluded_ - BC_original_).


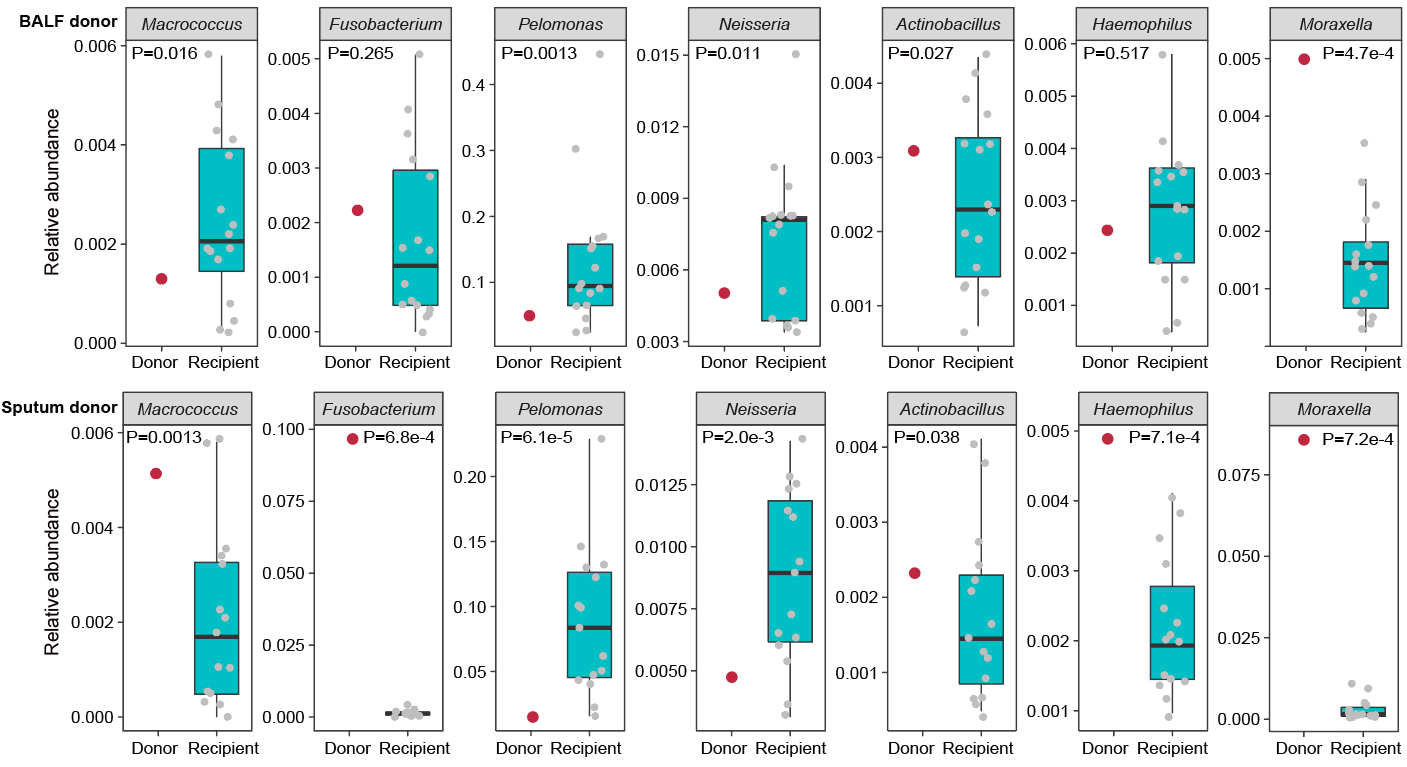


**Supplementary Fig. 2.** The relative abundance of seven bacterial genera (*Macrococcus*, *Fusobacterium*, *Pelomonas*, *Neisseria*, *Actinobacillus*, *Haemophilus*, and *Moraxella*) in donor (human bronchoalveolar lavage fluid [BALF] and sputum) and recipient mouse BALF samples in the human-to-mouse respiratory microbiota transplantation.

**Appendix 1. Protocol for respiratory microbiota transplantation.**

**Materials and reagents**

1. Physiological Saline Solution (Sichuan Kelun Pharmaceutical Co., Ltd., catalog number: B14200853444)
2. Phosphate Buffered Saline (PBS) (Beyotime Biotechnology, catalog number: C0221A)
3. Peracetic Acid (Guangzhou Ruisite Medical Technology Co., Ltd., catalog number: PD0028)
4. Glycerol (Millipore Sigma, catalog number: G7893)
5. Tribromoethanol (Avertin) (Sigma-Aldrich, catalog number: T48402 or CAS 75-80-9)
6. 75% Ethanol (Shandong Lierkang Medical Technology Co., Ltd.)
7. 1 mL Sterile Syringe (Shanghai Kindly Enterprise Development Group Co., Ltd., catalog number: 60017031)
8. Surgical Scissors (Shanghai Medical Instrument Co., Ltd. Surgical Instrument Factory, catalog number: Y00030)
9. Eye Forceps (Huasidic, catalog number: HKCL-20)
10. Indwelling Needle (Catheter) (B. Braun Melsungen AG, catalog number: 4254511-03)
11. Cotton Thread (Shenzhongshi Scientific Instruments Co., Ltd., catalog number: 10203)
12. 1.5 mL Centrifuge Tubes (Thermo Scientific Nunc, catalog number: 32195455321)
13. Brain Heart Infusion (BHI) Broth (Thermo Scientific, catalog number: CM1032B)
14. Tryptic Soy Broth (TSB) (Thermo Scientific, catalog number: CM1016T)
15. Columbia CNA Agar with Blood Plates (9 cm) (Qingdao Hi-Tech Industrial Park Haibo Biotechnology Co., Ltd., catalog number: HBPM0153)
16. Anaerobic Gas Packs (Mitsubishi, Guangzhou Kerong Biological Co., Ltd., catalog number: MG-C016)
17. Sterile Filters (0.22 µm) (Merck Millipore, catalog number: SLGP033R)
18. N-Acetyl-L-cysteine (NAC) (Macklin, catalog number: N80425)
19. Tracheal Intubation Cannula (Nanjing Calvin Biotechnology Co., Ltd., catalog number: KW-800)

**Equipment**

1. Centrifuge (Thermo Fisher Scientific, model: Thermo FRESCO 21)
2. -80 °C Ultra-low Temperature Freezer (Thermo Fisher Scientific, model: 902GP-ULTS)
3. Autoclave (High-Pressure Steam Sterilizer) (Shandong Xinhua Medical Instrument Co., Ltd., model for laboratory animals: BIST-A-D910-D-B)
4. Laboratory Homogenizer (Yilin/Huilian Instruments, model: FSH-2A)
5. Clean Bench (Laminar Flow Hood) (Suzhou Jingantai Air Technology Co., Ltd., AIRTECH, model: BCM-1000A)
6. Microbial Incubator (Thermo Fisher Scientific, model: IMH180)
7. pH Meter (Mettler Toledo, model: FE28)
8. Mouse Tracheal Intubation Station (Huaibei Dasijiaer Biological Technology Co., Ltd., catalog number: DS-CGT)

**Procedure**

**I. Sample collection and processing**

Human BALF collection and processing

Human BALF collection should follow standard safety and monitoring procedures for clinically indicated bronchoscopy. The upper respiratory tract, oral cavity, and lower respiratory tract are potential sources of the lung microbiome. The bronchoscope insertion route does not significantly affect BALF microbiota abundance. Transnasal bronchoscopy may minimize the possibility of upper respiratory microbiota contaminating the bronchoscope channel. The sampling method can be chosen based on clinical circumstances.

a. Collect BALF via clinical transoral or transnasal bronchoscopy. Avoid suction during bronchoscope insertion to prevent contamination from transferring the respiratory microbiome. Wedge the bronchoscope into the right middle lobe or left lingula. Perform bronchoalveolar lavage using sterile physiological saline (3–4 instillations of 50 mL each per patient), collecting approximately 130–300 mL of BALF. Remove 4 mL from this collection as the donor sample for transplantation. Typically, BALF from one patient is used for one recipient mouse; adjust the volume according to experimental needs.

b. Place the collected BALF on ice. Centrifuge at 2,500 × *g*, 4 °C for 7 min to pellet host cells. Retain the supernatant on ice (recommended storage time ≤ 3 hours).

c. Centrifuge the supernatant at 12,000 × *g*, 4 °C for 10 min to obtain the bacterial pellet. Resuspend the pellet in sterile PBS to 5% (w/v) (e.g., 1 mL PBS per 20 mg pellet estimated weight, adjust volume based on pellet size) aiming for a resuspended bacterial solution with OD600 = 0.5. This is the final lung bacterial sample for transplantation.

d. If the bacterial sample cannot be used immediately, add 20% sterile glycerol to the bacterial pellet from step 1c. Store at -80 °C (recommended storage duration: 2 weeks). Thaw frozen samples on ice. Centrifuge at 12,000 × *g*, 4 °C for 10 min to remove the glycerol supernatant. Retain the bacterial pellet. Resuspend each pellet in sterile PBS.
Note: The volume for resuspension in step 1c depends on the number of mice for transplantation, calculated at 50 µL per mouse.

Human sputum sample processing
Sputum is considered to consist of mucus plugs derived from the lower airways, often expectorated, collected, and analyzed to gain insights into the lung microbiome. All required consumables (EP tubes, pipette tips, glycerol, etc.) must be autoclaved and placed in a laminar flow hood.

a. For thin sputum samples: After collection, add 2 volumes of sterile PBS. Mix thoroughly by pipetting. Add glycerol to a final concentration of 10%. Samples can be stored long-term at -80 °C until use.

b. For viscous sputum samples: After collection, add 1 volume of N-acetylcysteine (NAC) for liquefaction. Centrifuge at 2,500 × *g* for 7 min. Discard the pellet and retain the supernatant.

c. Centrifuge the supernatant at 12,000 × *g*, 4 °C for 10 min. Retain the bacterial pellet.

d. Add 20% sterile glycerol to the bacterial pellet to resuspend it. Store at -80 °C (recommended storage duration: 2 weeks).

e. Thaw frozen samples on ice. Centrifuge at 12,000 × *g*, 4 °C for 10 min to remove glycerol. Retain the bacterial pellet. Add sterile PBS to the bacterial pellet for resuspension. The resuspension volume depends on the number of mice for transplantation, calculated at 50 µL per mouse.

Mouse BALF sample processing

a. Autoclave and dry surgical scissors, forceps, EP tubes, and other required equipment, then place them in a laminar flow hood. All mouse BALF collections should be performed under the hood to avoid contamination.

b. Use healthy adult mice as BALF donors. The volume for each lavage in mice is 500–800 µL. Typically, BALF from 2 donor mice is used for 1 recipient mouse; adjust according to experimental needs.

c. Anesthetize the mouse via intraperitoneal (IP) injection of 1.25% tribromoethanol (Avertin, 250 mg/kg) using a 1 mL syringe.

d. Disinfect the neck area with 75% ethanol. Use surgical scissors to cut the neck skin. Dissociate the salivary glands. Make a longitudinal incision along the sternohyoid muscle to expose the trachea. Place a cotton thread underneath the trachea. Make a small incision in the trachea.

e. Insert the indwelling needle (catheter) into the trachea approximately 0.5 cm (avoid inserting too deeply to prevent lung damage). Secure the needle and trachea with the cotton thread.

f. Aspirate 800 µL of ice-cold physiological saline into a 1 mL syringe. Attach it to the catheter. Perform three cycles of instillation and aspiration. Collect the recovered lavage fluid in a 1.5 mL EP tube placed on ice. Repeat the aspiration process twice per animal, collecting lavage fluid three times total per mouse.

g. Pool the three consecutive BALF collections from each mouse. Centrifuge the pooled BALF at 12,000 × *g*, 4 °C for 10 min to obtain the bacterial pellet. Resuspend the pellet in sterile PBS to 5% (w/v) aiming for a resuspended solution with OD600 = 0.5. This is the final BALF transplant sample, which should ideally be used immediately.

h. If the BALF sample cannot be used immediately, add 20% sterile glycerol to the bacterial pellet obtained in step 3g. Store at -80 °C (recommended storage duration: 2 weeks). Before use, thaw and resuspend each sample in an appropriate volume of sterile PBS (e.g., 2 mL, adjust based on pellet size and target concentration).

**II. Lung microbiome transplantation methods**

Intranasal instillation delivers bacterial suspension to both the upper and lower respiratory tract of mice and is often used for upper respiratory tract studies. A portion of the instilled bacteria may colonize the lungs, while the remainder may be absorbed through the nasal mucosa into the systemic circulation and colonize other organs. Intratracheal instillation is suitable for studies aiming to deliver bacteria directly to the lower respiratory tract and lungs, maximizing local effects and minimizing systemic absorption.

Intranasal Instillation in Germ-free Mice
a. Aseptically filter 1.25% tribromoethanol using a 0.22 µm sterile filter under the laminar flow hood. Transfer the sterile anesthetic, a 1 mL sterile syringe, and the bacterial sample (after external surface disinfection of packaging) into the isolator.

b. Anesthetize the mouse by intraperitoneal (IP) injection. Observe the mouse's breathing after anesthesia; successful anesthesia is indicated by the mouse entering a sleep state while maintaining a heartbeat.

c. Using the left hand, grasp the neck skin behind the ears of the GF mouse between the thumb and index finger. Secure the dorsal skin and tail with the ring finger and little finger, taking care to prevent suffocation. Align the head, neck, and body in a straight line, fully exposing the head.

d. Aspirate 50 µL of bacterial suspension into a 1 mL syringe. Connect the syringe to a tracheal cannula (22G) or blunt needle. Instill the bacterial solution drop by drop into the mouse's nostrils, allowing it to be inhaled automatically. Instillation is successful if the liquid is inhaled. Avoid instilling too rapidly.

Intratracheal Instillation in Germ-free Mice
a. Aseptically filter 1.25% tribromoethanol using a 0.22 µm sterile filter under the laminar flow hood. Transfer the sterile anesthetic, a 1 mL syringe, and the bacterial sample (after external surface disinfection of packaging) into the isolator.

b. Aspirate 50 µL of bacterial suspension into a 1 mL syringe. Replace the needle with a tracheal cannula (22G) and set it aside.

c. Anesthetize the mouse via IP injection of 1.25% tribromoethanol (250 mg/kg). After anesthesia, position the mouse supine and secure it on the intubation animal workstation.

d. Fix the mouse's incisors using the incisor ring on the workstation. Secure the forelimbs with tape. Slowly tilt the workstation to a 45-degree angle.

e. Illuminate the mouse's oral cavity from outside the isolator using a headlamp. Use forceps to lift the tongue upward and secure it with a spatula, making the trachea clearly visible.

f. Observe the glottis opening and closing with breathing. At the moment the glottis opens, slowly insert the tracheal cannula into the trachea.

g. After successful intubation, inject the 50 µL bacterial suspension slowly through the cannula, ensuring all liquid enters the lungs. Successful instillation is confirmed by bubbles appearing in the trachea upon withdrawal of the cannula needle.

h. Keep the mouse in an upright position on the workstation for 1 minute after the procedure. Slowly return the workstation to a flat position. Return the mouse to its housing cage.

**III. Assessment of lung microbiota colonization**

Post-transplantation, colonization efficiency can be confirmed by collecting BALF and lung tissue samples from recipient mice for microbial culture or molecular diagnostic techniques. Compared to conventional PCR, qPCR or ddPCR offers lower detection limits and a wider dynamic range, making it suitable for quantifying bacterial DNA and analyzing strain differences in samples with low bacterial abundance (gene copy number < 1e5). Bacterial DNA was extracted from transplanted mouse BALF or lung tissue samples. PCR amplification was performed using primers targeting the 16S rRNA gene. Amplicon sequencing can be conducted targeting the 16S rRNA gene hypervariable regions (i.e. V4 region) for microbiota analyses. Microbial biomass is often low for BALF, sputum or lung tissue samples, making them susceptible for reagent contaminations. Be sure to include reagent controls for DNA extraction and PCR amplification and be cautious of contaminating DNA during the entire procedure of sample processing and sequencing.
